# Deep learning performance for detection and classification of microcalcifications on mammography

**DOI:** 10.1186/s41747-023-00384-3

**Published:** 2023-11-07

**Authors:** Filippo Pesapane, Chiara Trentin, Federica Ferrari, Giulia Signorelli, Priyan Tantrige, Marta Montesano, Crispino Cicala, Roberto Virgoli, Silvia D’Acquisto, Luca Nicosia, Daniela Origgi, Enrico Cassano

**Affiliations:** 1 Breast Imaging Division, IEO European Institute of Oncology IRCCS, Milan, Italy; 2 Department of Radiology, King’s College Hospital NHS Foundation Trust, London, UK; 3 Laife Reply, Milan, Italy; 4 Medical Physics Unit, IEO European Institute of Oncology IRCCS, Milan, Italy

**Keywords:** Artificial intelligence, Machine learning, Mammography, Microcalcifications, Neural networks (computer)

## Abstract

**Background:**

Breast cancer screening through mammography is crucial for early detection, yet the demand for mammography services surpasses the capacity of radiologists. Artificial intelligence (AI) can assist in evaluating microcalcifications on mammography. We developed and tested an AI model for localizing and characterizing microcalcifications.

**Methods:**

Three expert radiologists annotated a dataset of mammograms using histology-based ground truth. The dataset was partitioned for training, validation, and testing. Three neural networks (AlexNet, ResNet18, and ResNet34) were trained and evaluated using specific metrics including receiver operating characteristics area under the curve (AUC), sensitivity, and specificity. The reported metrics were computed on the test set (10% of the whole dataset).

**Results:**

The dataset included 1,000 patients aged 21–73 years and 1,986 mammograms (180 density A, 220 density B, 380 density C, and 220 density D), with 389 malignant and 611 benign groups of microcalcifications. AlexNet achieved the best performance with 0.98 sensitivity, 0.89 specificity of, and 0.98 AUC for microcalcifications detection and 0.85 sensitivity, 0.89 specificity, and 0.94 AUC of for microcalcifications classification. For microcalcifications detection, ResNet18 and ResNet34 achieved 0.96 and 0.97 sensitivity, 0.91 and 0.90 specificity and 0.98 and 0.98 AUC, retrospectively. For microcalcifications classification, ResNet18 and ResNet34 exhibited 0.75 and 0.84 sensitivity, 0.85 and 0.84 specificity, and 0.88 and 0.92 AUC, respectively.

**Conclusions:**

The developed AI models accurately detect and characterize microcalcifications on mammography.

**Relevance statement:**

AI-based systems have the potential to assist radiologists in interpreting microcalcifications on mammograms. The study highlights the importance of developing reliable deep learning models possibly applied to breast cancer screening.

**Key points:**

• A novel AI tool was developed and tested to aid radiologists in the interpretation of mammography by accurately detecting and characterizing microcalcifications.

• Three neural networks (AlexNet, ResNet18, and ResNet34) were trained, validated, and tested using an annotated dataset of 1,000 patients and 1,986 mammograms.

• The AI tool demonstrated high accuracy in detecting/localizing and characterizing microcalcifications on mammography, highlighting the potential of AI-based systems to assist radiologists in the interpretation of mammograms.

**Graphical Abstract:**

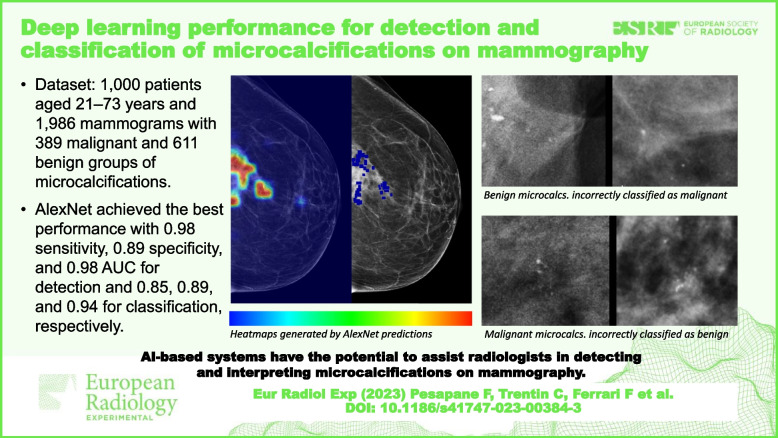

**Supplementary Information:**

The online version contains supplementary material available at 10.1186/s41747-023-00384-3.

## Background

Breast cancer is the most diagnosed cancer among women worldwide, and early detection is crucial for successful treatment and higher survival rate [1]. Although mammography remains the primary screening test that has demonstrated a reduction in breast cancer-related mortality, the utilization of mammography has shortcomings that pose challenges to its effectiveness and efficiency [2–4]. The large amount of mammograms produced every year, coupled with scarcity of trained radiologists capable of interpreting these exams, poses a risk the studies are reported poorly and with delays, losing the window of opportunity for optimal clinical intervention [5]. An overloaded screening service could become inefficient, leading to additional economical costs and inequalities between low- and high-income countries [6].

The introduction of artificial intelligence (AI) in medical image analysis has brought forth a potential revolution in computer-based interpretation of mammography [4, 5, 7].

AI introduction implies technological, ethical, and legal considerations (especially around data privacy and AI influence on medical liability) and different patient’s perspectives on AI integration, ranging from cautious support to concerns about overreliance and potential loss of human interaction [7, 8]. However, it has the potential to revolutionize the field by addressing the limitations of mammography interpretation and improving breast cancer diagnosis. AI-based tools hold the potential to reduce the time invested by radiologists in scrutinizing mammography screening images, offering the capacity to identify and characterize abnormalities present on mammograms. Radiologists could proceed faster through cancer-free cases and give more attention to the images with suspicious findings. Shifting time allocation and integrating AI for mammogram interpretation could enhance cost-effective accessibility to screening worldwide, particularly aiding low- and middle-income countries facing equipment costs and expertise limitations while also tackling radiologist shortages in high-income countries to ensure successful breast cancer screening [5, 9].

Mammography provides a rich domain for scalable clinical AI application. Several studies are currently evaluating how and when AI will be successfully used in clinical practice [10–14]. Particularly, AI systems may have clinical value for early detection and treatment of breast cancer—by filtering out cancer-free mammograms, resulting in lower recall rates and reducing the number of unnecessary biopsies [15–18]. This would free up time for managing suspected and proven cancers and optimize clinical interventions. Moreover, AI has the potential to surpass current techniques for the detection and classification of breast microcalcifications [19–22], namely tiny calcium deposits that can be an early sign of breast cancer. While mammography is the primary imaging tool for microcalcifications assessment, their detection and discrimination can be challenging and subjective for radiologists, leading to high interobserver variability [23]. Deep learning (DL) tools can alleviate some diagnostic challenges, improving breast cancer screening accuracy and reducing the need for unnecessary biopsies.

Therefore, the development of a standardized, observer-independent microcalcifications detection and categorization system is warranted. Once trained, the tool can then analyze new mammograms, accurately detect and classify microcalcifications, providing radiologists with a second opinion to improve diagnostic accuracy.

In this context, our study aims to develop a deep DL-based network, with the following aims:

- Task 1, to detect and localize suspicious microcalcifications in digital mammography
- Task 2, to accurately classify microcalcifications into benign or malignant categories

## Methods

### Ethics statement

The Institutional Review Board of the European Institute of Oncology (IEO) approved this study: protocol UID 3052 and date of approval 7 October 2021.

### Patient population and dataset

The dataset was collected at a single institution, which is an academic hospital and referral centre for breast cancer care. All patients had an interval of less than 1 month between vacuum-assisted breast biopsy and the diagnostic mammography.

The patient selection criteria included individuals who had undergone and subsequently had diagnostic mammography performed within an interval of less than 1 month.

The dataset contained a total of 1,986 mammography images from 1,000 patients with age 45 ± 10 years (mean ± standard deviation), ranging 21–73 years, including 611 benign lesions and 389 histologically proven breast cancers. Accordingly, the pathological analysis through needle biopsy or surgery was the ground truth reference standard of the microcalcifications included in the region of interest (ROI). To be representative of the population that the model will be applied to, we selected heterogeneous data of patients including women with different breast densities — according to the American College of Radiology (ACR) classification [24] — medical histories, and demographics data, as reported in the “Results” section. To maintain data quality, we carefully excluded any poor-quality mammography images, such as those with low resolution.

Each mammography image in the dataset was meticulously annotated by three expert radiologists from the breast imaging department of a national referral centre for breast cancer care [25]. These annotations served to localize the microcalcifications within the images and provide valuable information on their benign or malignant characteristics.

### Study design and workflow

**Fig. 1:**
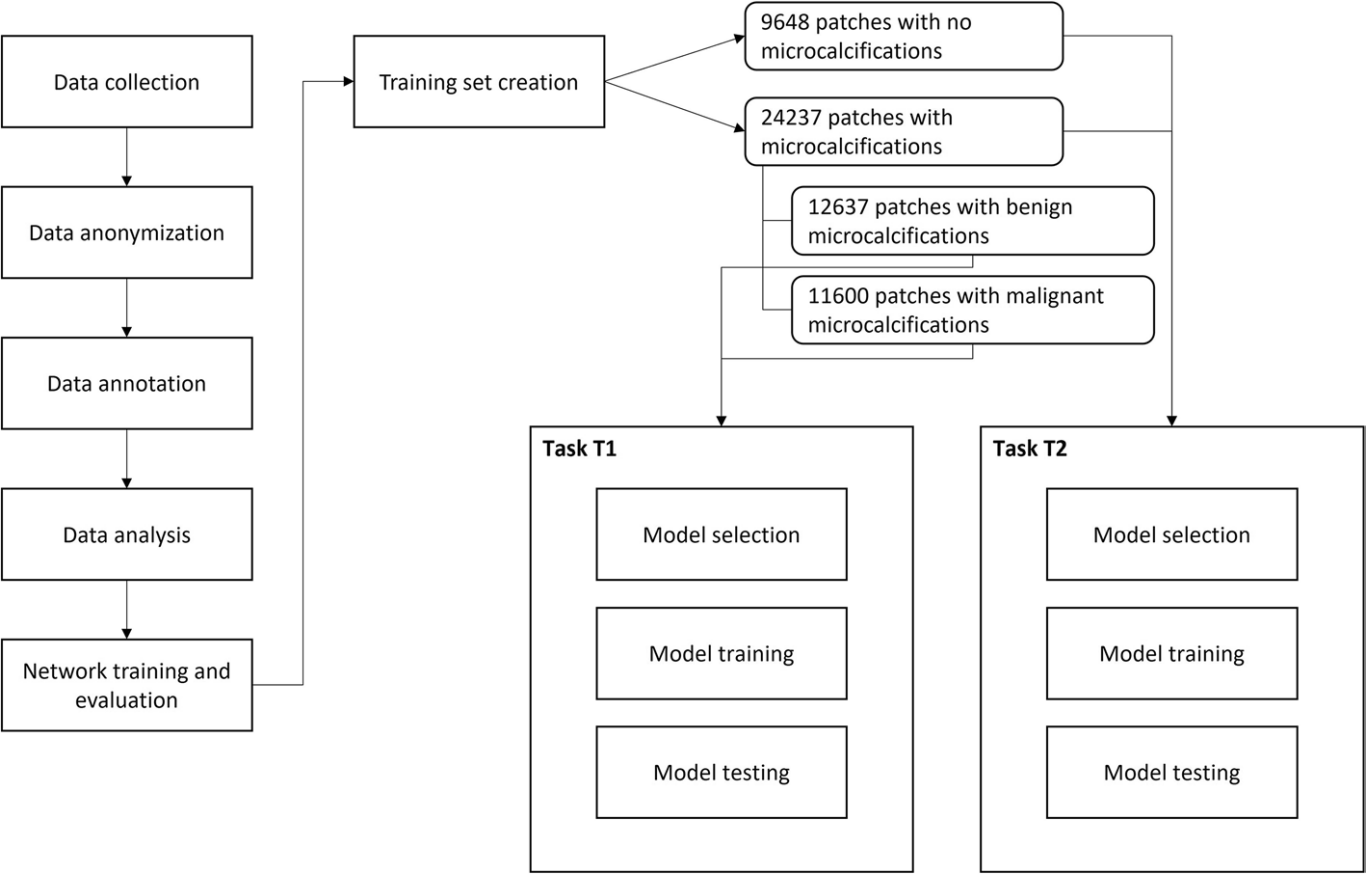
Study flowchart illustrating the workflow of the study, highlighting the key steps involved in the analysis of mammographic images for microcalcifications classification. The study follows a 5-point framework, encompassing data collection, anonymization, annotation, analysis of annotated data, and network training and evaluation. The flowchart provides a visual representation of the interplay between these phases and the various patient subdivisions, including training, validation, and testing. Numbers of patches are indicated to convey the distribution of microcalcifications and their nature (benign/malignant)

**Fig. 2:**
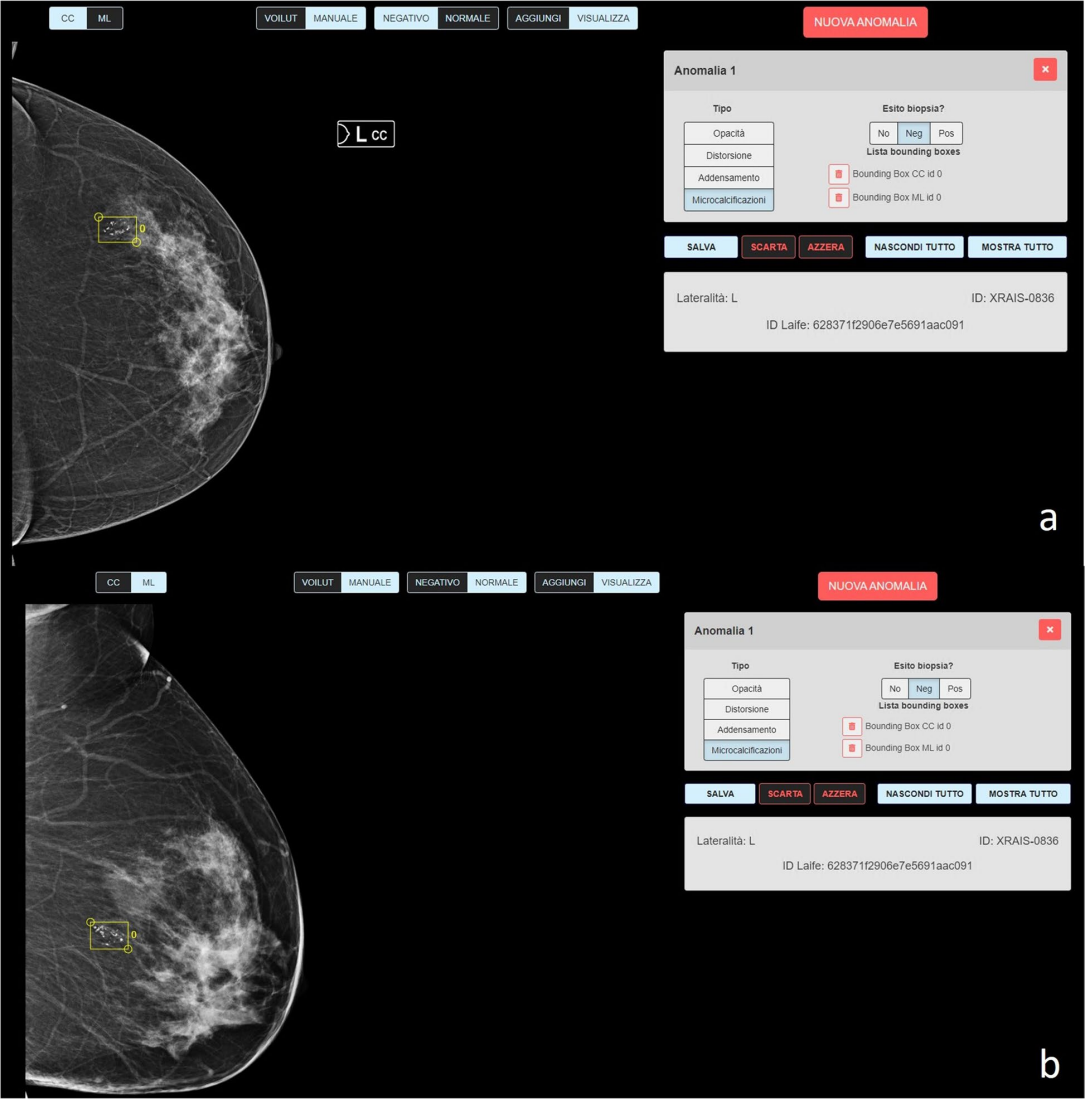
Example of annotations of suspicious microcalcifications in craniocaudal (**a**) and medio-lateral (**b**) mammograms performed by radiologists using a special application for tagging (X-RAIS, see the “Methods”)

The workflow of the study included the following five different phases, as shown in the flowchart (Fig. 1):

1. Data collection: Two radiologists retrospectively collected cases from a pool of patients who meet the inclusion criteria, namely mammography exams performed at a single institute (European Institute of Oncology, Milan, Italy) containing microcalcifications for which the histology result was available.
2. Anonymization: The selected images, in DICOM format, were made completely anonymous during the extraction phase.
3. Annotation: Three expert radiologists annotated the selected cases. During the annotation phase, the radiologists used a special application made available by Laife Reply, namely the X-RAIS tagging tool [26], which made it possible to correlate the location of suspicious microcalcifications for each image and the binary classification of benign/malignant, as reported by the outcome of the histological investigation. Figure 2 shows an example of annotation of suspicious microcalcifications by a radiologist.
4. Analysis of annotated data: Data scientists verified the consistency of the annotations made on the images for the purposes of DL algorithm training.
5. Networks training and evaluation: Using the annotated data, the data scientists trained convolutional neural networks (CNNs) to analyze mammographic images towards the two aims of the study.

### Training set creation

The dataset contains 1,986 full-field digital mammograms, with resolutions variable from 1,912 × 2,294 to 4,700 × 5,800 and a depth of 12 bit. Since the annotated images were too large to be used in their original size, a patch approach was used [22]. The rationale is to extract, from the original images, the portions containing the areas noted by radiologists or tissue without microcalcifications. Particularly, the criterion for extracting the annotated areas starts from the central point of a single annotation and defines a ROI of size 112 × 112 pixels around it.

The ROI was then saved and considered from this moment on a patch of “microcalcifications” that, depending on the annotation, could be benign or malignant. Supplementary Fig. S1 and Supplementary Fig. S2 show an example of benign and malignant microcalcifications, respectively.

For the extraction of “non-microcalcifications” patches, the same images are reused, but a different extraction criterion was applied, aimed at obtaining a set composed only of tissue patches without microcalcifications. To guarantee the heterogeneity of the dataset, the number of non-microcalcifications patches to be extracted is similar to the number of microcalcifications patches.

To extract non-microcalcifications patches, a point of the image is randomly chosen, and an area of interest of 112 × 112 pixel is built around it. The patch's inclusion is contingent upon satisfying two criteria: first, an average color intensity surpassing a specified threshold (implemented to prevent the extraction of patches unrelated to the tissue); second, avoidance of overlap with annotated regions.

At the end of the dataset creation procedure, we obtained the following: 24,237 patches with microcalcifications (12,637 benign and 11,600 malignant) and 9,648 non-microcalcifications patches.

The dataset was partitioned by assigning 70% of the patches to the training set, 20% to the validation set, and 10% to the test set. Particularly, after splitting, we obtained the following:

- Task 1 (suspect microcalcifications *versus* non-microcalcifications)
  - Train split, 23,730 patches, 71% microcalcifications and 29% non-microcalcifications
  - Validation split, 6,789 patches, 71% microcalcifications and 29% non-microcalcifications
  - Test split, 3,393 patches, 71% microcalcifications and 29% non-microcalcifications
- Task 2 (benign *versus* malignant)
  - Train split, 16,977 patches, 52% benign and 48% malignant
  - Validation split, 4,859 patches, 53% benign and 47% malignant
  - Test split: 24,26 patches, 54% benign and 46% malignant

Notably, the extracted patches were carefully allocated to different subsets during the training process, thereby mitigating the potential issue of reusing the same image portions for both training and validation/testing phases. To accomplish this, a methodical approach to grouping was adopted, specifically designed to safeguard against the separation of patches originating from the same image and images derived from the same patient (attributable to the inclusion of dual views per patient) into different subsets. This division of patches aimed to eliminate any possibility of artificially inflated performance by guaranteeing that no identical image regions were utilized across training, validation, and testing sets.

### Model selection

After a review of the state of the art [19, 22, 23, 27–29], we have selected a small set of networks, namely three CNNs, to be used for their training. In particular, the three networks chosen were the following: AlexNet, ResNet18, and ResNet34. Using these networks, we evaluated whether increasing the complexity of the networks corresponded to better performance. For each of the above-mentioned neural networks, it was necessary to make a variation of the last level, to have only two exit nodes, necessary to carry out a binary classification task.

### Model training

As reported before, the two tasks of this study are as follows:

- Task 1, classification of patches containing microcalcifications or not containing microcalcifications
- Task 2, classification of benign *versus* malignant microcalcifications patches

The training data used in Task 2 is therefore limited to microcalcifications patches only, while training data used in Task 1 includes also non-microcalcifications patches. Accordingly, the localization of microcalcifications was performed through a classification network, whose input is patches extracted from the original image following a sliding window approach. Finally, the dataset underwent pre-processing operations (blur, normalization, and resize) which allowed obtaining better results.

Hyperparameter tuning was performed on each neural network, alongside with early stopping to avoid overfitting. Particularly, following rigorous evaluation, all proposed models were trained from the ground up, employing a cross-entropy loss function, the Adam optimizer, a batch size of 16, and a learning rate of 0.0001.

### Predictions interpretability

**Fig. 3:**
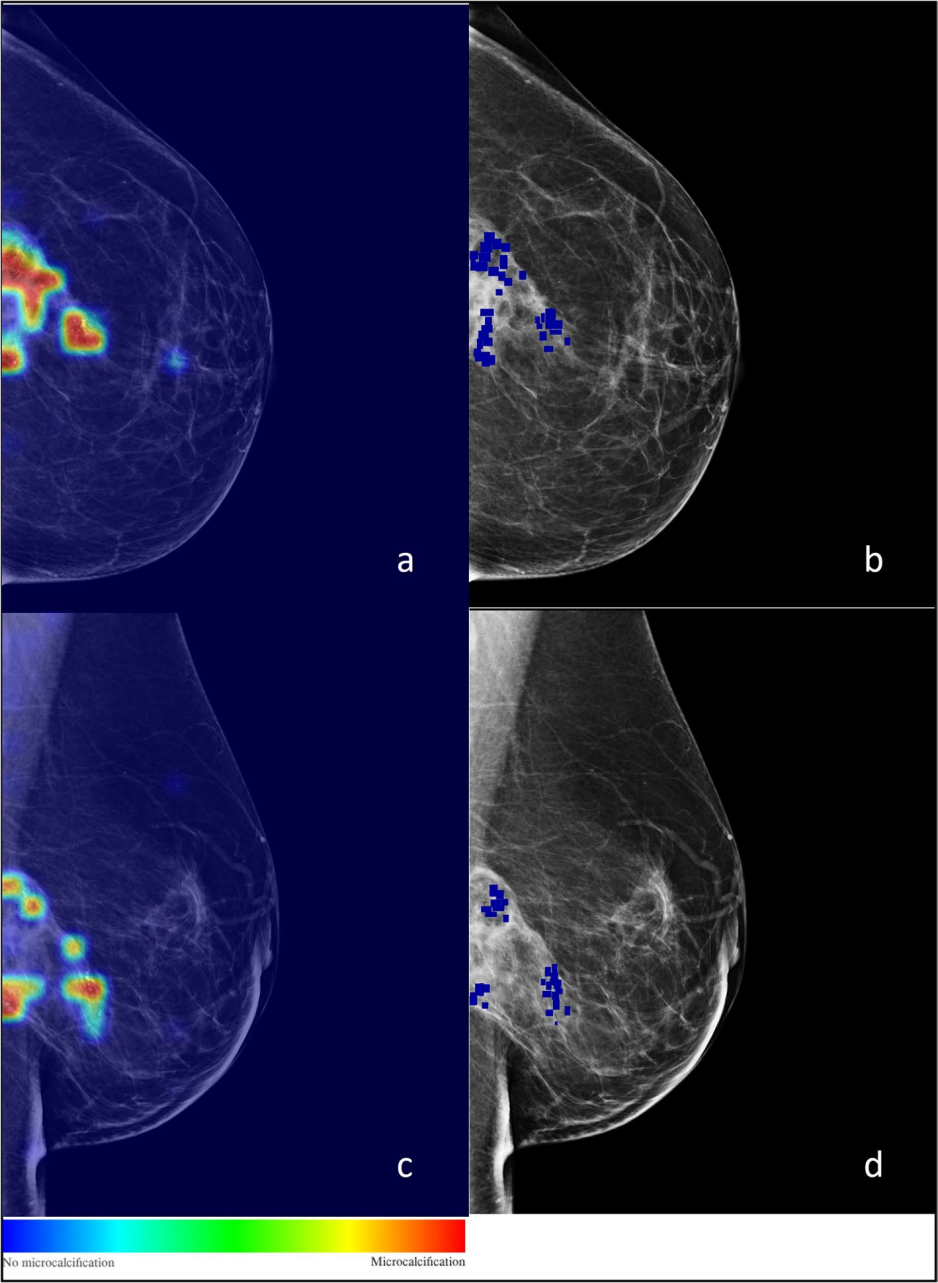
Example of heatmaps generated by Task 1 AlexNet predictions (**a**, **c**), compared with radiologists’ annotations (**b**, **d**). Colour changes based on the probability of the predictions. The colour scale visually represents the probability of microcalcifications in the area; it ranges from blue to red, which are 0% and 100%, respectively

To evaluate the interpretability, we performed a visual assessment of the heatmaps generated from the individual patches. The generation of heatmaps in this study involved a multistep process. Initially, the entire mammogram was partitioned into nonoverlapping patches using a sliding window technique. Each patch was then individually processed through the trained model, which assigned a probability value representing the likelihood of that patch containing microcalcifications. Subsequently, a grid was constructed, where each cell of the grid corresponded to a specific patch and contained the probability associated with the presence of microcalcifications. By the end of the processing, this grid served as a mask, which was superimposed on the mammogram to create a heatmap. The heatmap visually represented the regions with the highest probabilities, indicated as hot patches, suggesting the areas that are most likely to contain microcalcifications. This technique provided a valuable tool for enhancing the interpretability and localization of microcalcifications within the mammogram, facilitating the identification and analysis of potential breast abnormalities. Figure 3 shows an example of a heatmap generated by model predictions, compared with annotations by radiologists: this information can help us understand the features or patterns that the CNN is utilizing to make its predictions.

### Model evaluation and statistical analysis

We evaluated the diagnostic performances of DL-based networks using various metrics, including positive predictive value (PPV), negative predictive value (NPV), sensitivity, specificity, diagnostic accuracy, and area under the curve at receiver operating characteristics (AUC). Descriptive statistics were computed to summarize the age distribution of the patient population.

Model metrics are reported with 95% confidence intervals (CIs) computed using the following formula:

$$\left[x-1.96\times \sqrt{\frac{x\times \left( 1-x\right)}{n}}, x+1.96\times \sqrt{\frac{x\times \left( 1-x\right)}{n}}\right]$$

where *x* is the metric and *n* is the size of the test set and then rounded.

We assessed the normality assumption of the age variable using the Shapiro–Wilk test. Calculations were performed using a specifically created Python script.

## Results

The dataset contained a total of 1,986 mammography images from 1,000 patients with age 45 ± 10 years (mean ± standard deviation), ranging 21–73 years. Mammographic breast parenchymal density was categorized as almost entirely fat (ACR category A) in 180 (18%), scattered fibroglandular tissue (ACR category B) in 220 (22%), heterogeneously dense (ACR category C) in 380 (38%), and extremely dense (ACR category D) in 220 (22%) patients. Patient’s medical histories also vary, with 350 (35%) patients having a family history of breast cancer, 110 (11%) patients with previous personal history of breast cancer, 120 (12%) patients had previous breast biopsies, and 75 (7.5%) had previous radiation therapy for breast cancer Table 1.

**Table 1:** Number of mammograms with malignant and benign breast findings

Histopathology result	Number
*Malignant*	
Invasive ductal carcinoma	132
Invasive lobular carcinoma	111
Triple-negative breast cancer	9
Inflammatory breast cancer	17
Ductal carcinoma *in situ*	120
Total	389
*Benign*	
Fibrocystic mastopathy	310
Granulomatous inflammation	8
Postsurgical scar	8
Fat necrosis	18
Fibroadenoma	7
Diffuse cellular stroma	88
Ductal papilloma	21
Ductal cell hyperplasia	92
Sclerosing adenosis	59
Total	611

Table 2 shows the performance results of all three networks at the end of the experimental study, with accuracy and AUC being the most relevant metrics. For Task 1, AlexNet showed the best performance, with an accuracy on the test set of 0.95 (95% CI 0.94–0.96), and an AUC on the test set of 0.98, reaching higher values than for Task 2. The AlexNet network was also the best in terms of sensitivity 0.98 (95% CI 0.98−0.98) and NPV 0.94 (95% CI 0.93–0.95), while the ResNet18 model showed the best results in terms of specificity 0.91 (95% CI 0.90–0.92) and PPV 0.96 (95% CI 0.95–0.97). For Task 2, the network that obtained the best results was again the AlexNet, with an accuracy on the test set of 0.87 (95% CI 0.86–0.88), and an AUC on the test set of 0.94. Sensitivity was 0.85 (95% CI 0.84–0.86), specificity was 0.89 (95% CI 0.88–0.90), PPV was 0.87 (95% CI 0.86–0.88), and NPV was 0.88 (95% CI 0.87–0.89). The results are reported at the patch level, meaning that the accuracy of 0.87 represents the performance of the network in correctly classifying individual patches within the test set.

**Table 2:** Final results of deep learning networks performance

Task	Network	AUC	Accuracy	Sensitivity	Specificity	PPV	NPV
1	AlexNet	**0.98**	**0.95 (0.94**−**0.96)**	**0.98 (0.98−0.98)**	0.89 (90.88−0.90)	0.96 (0.95−0.97)	**0.94 (0.93−0.95)**
	ResNet18	0.98	0.95 (0.94−0.96)	0.96 (0.95−0.97)	**0.91 (0.90−0.92)**	**0.96 (0.95−0.97)**	0.91 (0.90−0.92)
	ResNet34	0.98	0.95 (0.94−0.96)	0.97 (0.96−0.98)	0.9 (0.89−0.91)	0.96 (0.95−0.97)	0.91 (0.90−0.92)
2	AlexNet	**0.94**	**0.87 (0.86−0.88)**	**0.85 (0.84−0.86)**	**0.89 (0.88−0.90)**	**0.87 (0.86−0.88)**	**0.88 (0.87−0.89)**
	ResNet18	0.88	0.80 (0.78−0.82)	0.75 (0.73−0.77)	0.85 (0.84−0.86)	0.80 (0.78−0.82)	0.80 (0.78−0.82)
	ResNet34	0.92	0.84 (0.83−0.85)	0.84 (0.83−0.85)	0.84 (0.83−0.85)	0.81 (0.79−0.83)	0.87 (0.86−0.88)

95% confidence intervals in parentheses. Best performance in bold. *AUC* Area under the curve, *NPV* Negative predictive value, *PPV* Positive predictive value

**Fig. 4:**
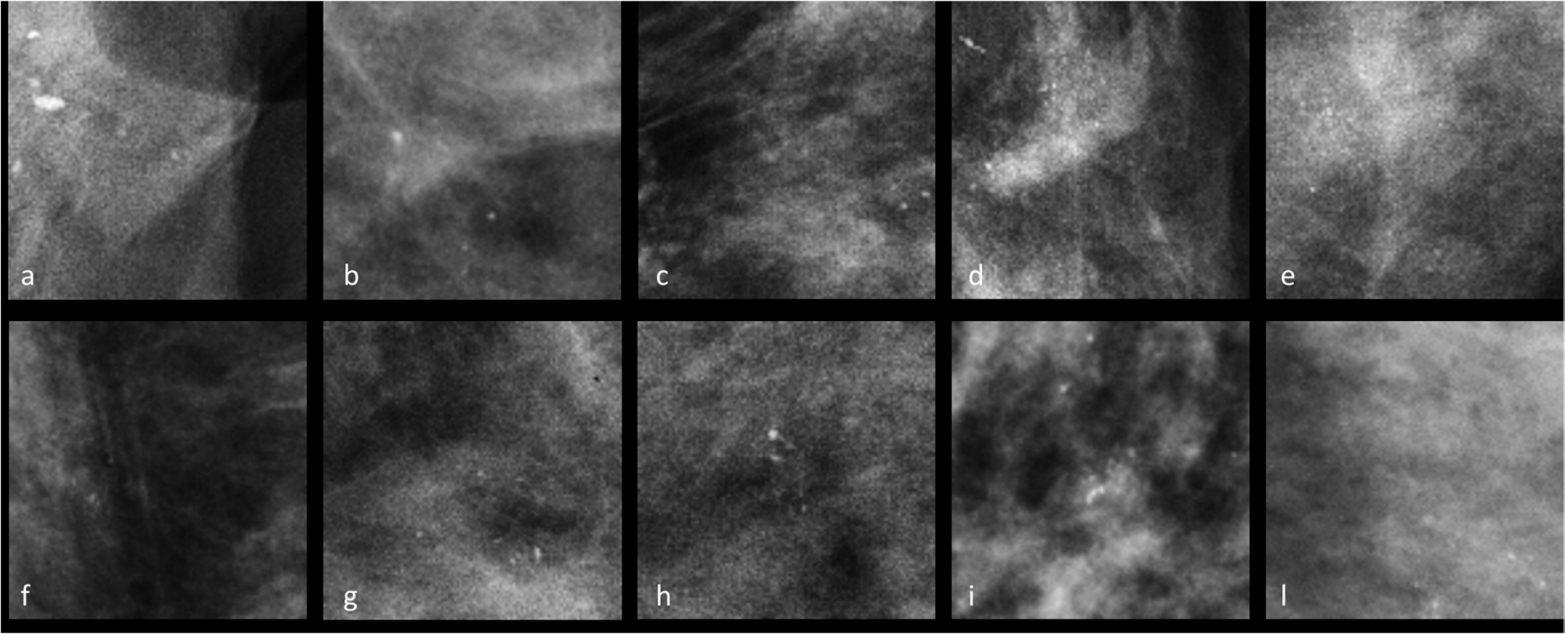
Examples of benign microcalcifications (**a**–**e**) incorrectly classified cases as malignant and malignant microcalcifications (**f**–**l**) incorrectly classified as benign

Figure 4 shows examples of microcalcifications that were incorrectly classified as either benign or malignant by the tested neural networks. This figure aims to visually demonstrate instances of misclassifications encountered by the CNNs, which are reflective of errors that expert human radiologists may also encounter during their clinical practice. These examples serve to emphasize the challenges faced by both automated systems and human observers when classifying microcalcifications accurately.

**Fig. 5:**
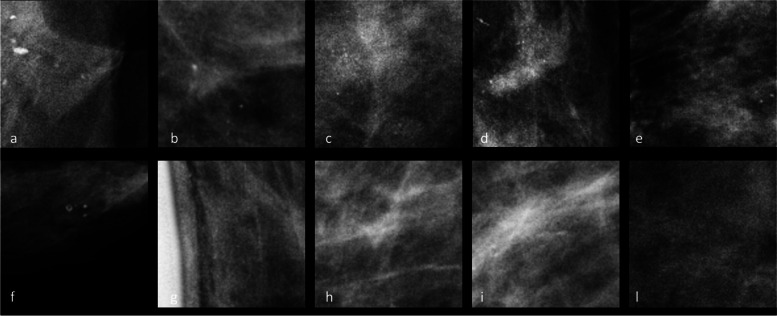
Examples of areas with microcalcifications (**a**–**e**) incorrectly classified cases as areas without microcalcifications and areas without microcalcifications (**f**–**l**) incorrectly classified as areas with microcalcifications

In addition, Fig. 5 shows areas with and without microcalcifications that were incorrectly classified by the tested neural networks. This figure further highlights the complexity of the task and the potential difficulties faced in distinguishing between regions with and without microcalcifications. As part of future studies, the incorporation of supplementary data could potentially aid in mitigating such instances of misclassification.

## Discussion

In this study, we evaluated the performance of CCNs in a dataset that included 1,986 mammography images from 1,000 patients, reflecting diverse demographics. The CCNs results showed AlexNet excelled in detecting and localizing microcalcifications for accuracy, AUC, sensitivity, and NPV, while ResNet18 performed best in specificity and PPV. In the characterization of microcalcifications, AlexNet again led in accuracy and AUC. These findings indicate that AI models can be trained to successfully diagnose malignant microcalcifications and identify mammograms devoid of microcalcifications.

Radiologists are already familiar with computer-aided detection systems, which were first introduced in the 1960s for mammography [30]. However, advances in algorithm development, combined with the ease of access to computational resources, allow AI to be applied in radiological decision-making at a higher functional level, achieving a sensitivity from 0.56 to 0.82 with a specificity of 0.84–0.97 [31, 32], comparable with breast cancer detection accuracy of radiologists [33].

In mammography, DL models can be trained on a large dataset of images, including those with microcalcifications, to learn patterns and features that are characteristic of benign and malignant microcalcifications. However, there is a lack of standardized approaches for data collection, annotation, and evaluation. This hinders the comparability of results from different studies and makes it challenging to establish a consensus on best practices. Our study contributes by following a well-defined workflow for data collection, annotation, and evaluation, ensuring robustness and reproducibility of the results. It was conducted in a cancer referral centre by radiologists with high experience in breast cancer indeed, which ensures that the tool was developed in a context of expert knowledge and clinical expertise. This also increases the likelihood that the tool will be applicable and relevant to real-world clinical practice. While AI models have shown promising results in research settings, their integration into real-world clinical practice poses practical challenges indeed. Implementing AI tools effectively requires seamless integration with existing clinical workflows and regulatory approval, as well as demonstrating their clinical utility.

One of the major challenges with AI models in mammography is the lack of interpretability and transparency in their decision-making process as CNNs often act as a “black box”, making it difficult to understand the features or patterns they use to arrive at a diagnosis [7]. Our study addresses this challenge by incorporating heatmap visualization to provide interpretability and transparency to the CNN’s decision-making process, enabling radiologists to understand the regions of interest considered by the AI for classification. As shown in Fig. 3, we use the heatmap to provide interpretability and transparency to CNN’s decision-making process. By analysing the heatmap, we can identify the specific regions of interest that the CNN considers when classifying microcalcifications as benign or malignant. This information can help us understand the features or patterns that CNN is utilizing to make its predictions. Furthermore, by examining the heatmap of misclassified cases (Figs. 4 and 5), we can gain insights into the potential limitations and challenges faced by CNN: analysing the heatmap can reveal whether the misclassifications occurred due to the presence of subtle or atypical features, the difficulty in distinguishing certain patterns, or any other factors that may contribute to classification errors indeed. This analysis can shed light on the areas where CNN may require further improvements or additional training data to enhance its performance.

Moreover, we tested the DL networks in a heterogeneous database of population, which increases the generalizability of the results. By including a diverse population, the tool’s ability to detect and classify microcalcifications in a wide range of breast tissue types and patient demographics can be evaluated, providing a more comprehensive assessment of its effectiveness.

Regarding the results of our study, high sensitivity and specificity were observed for Task 1 and Task 2, confirming that the proposed CNNs correctly detect and classify microcalcifications, without showing imbalances towards one of the two tasks. Among the three different networks, AlexNet obtained the best results for Task 1 and Task 2 (see Table 2). Specifically, results of Task 2 demonstrate a high capacity of the model to discriminate between the benign and malignant microcalcifications. Furthermore, the results of the ResNet18 and ResNet34 networks show that increasing the complexity of the network does not correlate with an increase in performance in terms of accuracy and AUC.

Once our results will be confirmed in a prospective validation study, the proposed DL-based tool could significantly reduce the time and variability associated with manual detection and discrimination of microcalcifications, leading to earlier diagnosis and treatment. It can also reduce the need for additional diagnostic tests, such as biopsy or second-level examinations like breast MRI or contrast-enhanced mammography, therefore limiting patient discomfort and cost.

So far, few studies [20, 22, 23] found that AI could help to characterize breast microcalcifications, and the novelty of our study lies in the integration of CNNs to address the challenges in not only microcalcifications classification but also their localization. By achieving these aims, our study seeks to contribute to the field by providing a standardized and reliable method for the observer-independent detection and categorization of microcalcifications. While studies have already demonstrated AI promises, more real-world evaluations and studies are crucial to fully understand its impact. Particularly, many AI models in mammography are trained and evaluated on relatively small datasets that might lack diversity in terms of patient demographics, breast tissue types, and medical histories [34]. As a result, the models may not generalize well to different populations and may exhibit bias. Our study takes a step towards addressing this issue by including a large and heterogeneous dataset representing a diverse patient population, which increases the generalizability of the AI tool.

Making a specific comparison of the results of our study with the state of the art is not feasible, since the dataset used is not public (just as the datasets used in other studies are often not public) and tasks do not always coincide. However, a few similar studies are reported which allow for a comparison with our results. Cai et al. [22] used a set of 3,564 ROIs extracted from 990 source images with 1,912 × 2,294 pixel size, and their results show the ability of the networks to distinguish patches of different types with 0.88 AUC, 0.93 sensitivity 0.88, and specificity 0.86. Accordingly, the results obtained, although with a different dataset, were consistent with the Task 2 of our study. Concerning Task 1, studies by Valvano et al. [28] and Alam et al. [29] showed similar performances, confirming the potential of using AI networks to localize areas containing microcalcifications on mammography.

Our DL-based tool has some limitations, especially for implementing it in clinical practice.

The first limitation is the limited size of the data. This study shows preliminary data, and the number of mammographic images analyzed is constantly increasing to better train AI networks. In the next phase, the amount of data for training, as well as for testing, will be expanded to achieve a higher accuracy. The retrospective testing on internal or external datasets was essential for assessing our new AI tool for clinical imaging [35]. It is paramount to distinguish between testing that is conducted internally by the AI developers and externally by an independent institution. Accordingly, our experience is important as it combines external technological consultancy with an internal and independent database and data analysis.

In the next steps, external testing will limit bias and will also allow for the comparison of multiple algorithms with similar applications [32]. There is a danger of innate latent bias built into certain systems, especially if these have been developed on datasets that underrepresent certain populations (*i.e.*, with a lack of diversity of age or of breast density) and therefore lack the ability to generalize [32]. Accordingly, we selected the sample to be representative of the population that the model will be applied to, including patients with different demographics, breast densities, and medical histories. Moreover, as the quality of the data used to train the model is essential for the accuracy of the model, we provided high-quality annotation of data by expert breast radiologists, and we excluded poor quality data, such as low-resolution mammography to be sure that the quality is adequate. On the other hand, such high-quality data may not reflect the real clinical practice. Overall, our data was heterogeneous and representative of the population, structured, annotated, and ready to use, which is something limited, currently existing in only a small number of institutions [35].

Another limitation for implementation in clinical practice is that our AI networks are focused on microcalcifications only, while in clinical practice, when reading mammography, the radiologist considers different aspects in addition to microcalcifications, like radiopacities and architectural distortions. Moreover, radiologists rely heavily on the comparison against the contralateral and the prior breast image during their exam interpretation, while current AI networks are not capable of comparing images across time [23]. Additionally, to make the proposed DL-based tool complete for the current state of the art of mammography, the application of such tool also in tomosynthesis, and not only in 2D mammograms, is demanded.

Finally, we recognize that a more in-depth analysis of misclassified clusters could provide deeper insights into the model’s weaknesses and potential areas for improvement. The exploration of misclassified areas, as shown in Fig. 5, highlights the intricate nature of distinguishing between regions with and without microcalcifications. Incorporating additional data in future studies could offer an avenue to address and mitigate these misclassifications, enhancing the overall robustness of our model’s performance.

Despite such limitations, our study contributes to the field of breast cancer diagnosis by evaluating the performance of AI-based neural networks in accurately detecting, localizing, and characterizing microcalcifications on mammography. While there have been previous studies on computer-aided detection systems in mammography [22, 28, 29, 32], our research stands out in several key aspects.

Firstly, we employed advanced DL models, specifically AlexNet, ResNet 18, and ResNet34, which have demonstrated excellent performance in various computer vision tasks. These models were trained on a large dataset of mammography images with microcalcifications, to capture the patterns and features indicative of both benign and malignant microcalcifications. By utilizing these state-of-the-art DL models, our study represents a significant advancement in the application of AI for breast cancer diagnosis. Secondly, we conducted our study in a real-world clinical setting, involving experienced radiologists with high expertise in breast cancer [25]. This ensures that the AI tool was developed with expert knowledge and clinical relevance, increasing the likelihood of its applicability in clinical practice. The inclusion of a diverse population in our dataset further enhances the generalizability of our results, allowing for evaluation across various breast tissue types and patient demographics. While our focus in this study was on microcalcifications, we acknowledge the importance of considering other aspects such as masses and architectural distortions, as well as the ability to compare images across time. This recognition highlights the future direction of our research, as we strive to develop comprehensive DL models that encompass these additional aspects for a more holistic breast cancer diagnosis.

In conclusion, the current study demonstrates the potential of a DL-based tool to automate detection and discrimination of breast microcalcifications on mammography. The tool achieved high levels of accuracy, sensitivity, and specificity, indicating its potential for clinical use. Once validated, the proposed tool can significantly reduce the time and variability associated with traditional detection and discrimination of microcalcifications, leading to earlier diagnosis and treatment. Finally, this study holds significant implications for improving breast cancer diagnosis and has the potential to enhance the accuracy and efficiency of screening programs, ultimately leading to better patient outcomes. However, further development is required, and additional research is needed to validate the proposed tool on larger datasets and to evaluate its clinical utility.

### Supplementary Information

**Additional file 1: Supplementary Fig. S1.** Example of benign microcalcifications. Cranio-caudal (a) and medio-lateral (b) mammograms show benign microcalcifications (arrows) in the upper-external quadrant of the left breast of a 54 years-old woman. **Supplementary Fig. S2.** Example of malignant microcalcifications. Cranio-caudal (a) and medio-lateral (b) mammograms show suspicious microcalcifications (arrows) in the lower-external quadrant of the right breast of a 60 years-old woman. A vacuum-assisted breast biopsy was performed under stereotactic guidance and the histological exam results were invasive ductal carcinoma.

## Data Availability

The data that support the findings of this study are available from IEO-European Institute of Oncology, but restrictions apply to the availability of these data, which were used under license for the current study, and so are not publicly available. Data are however available from the authors upon reasonable request and with permission of the IEO-European Institute of Oncology.

## Abbreviations

ACR: American College of Radiology
AI: Artificial intelligence
AUC: Area under the curve at receiver operating characteristics analysis
CI: Confidence interval
CNNs: Convolutional neural networks
DL: Deep learning
NPV: Negative predictive value
PPV: Positive predictive value
ROI: Region of interest
