# Supplementary material for: Deep learning performance for detection and classification of microcalcifications on mammography
Source: Eur Radiol Exp. 2023 Nov 7;7:69. doi: 10.1186/s41747-023-00384-3 (PMC10630180; doi:10.1186/s41747-023-00384-3)
Supplement: Supplementary file 1 — Additional file 1: Supplementary Fig. S1. Example of benign microcalcifications. Cranio-caudal (a) and medio-lateral (b) mammograms show benign microcalcifications (arrows) in the upper-external quadrant of the left breast of a 54 years-old woman. Supplementary Fig. S2. Example of malignant microcalcifications. Cranio-caudal (a) and medio-lateral (b) mammograms show suspicious microcalcifications (arrows) in the lower-external quadrant of the right breast of a 60 years-old woman. A vacuum-assisted breast biopsy was performed under stereotactic guidance and the histological exam results were invasive ductal carcinoma. [file 41747_2023_384_MOESM1_ESM.docx]

**Deep learning performance for detection and classification of microcalcifications on mammography**

**ELECTRONIC SUPPLEMENTARY MATERIAL**

**
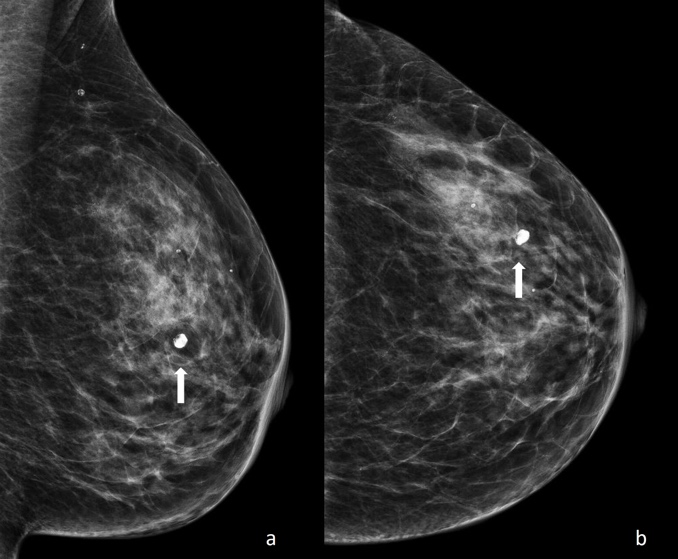
**

**Supplementary Fig. S1.** Example of benign microcalcifications. Cranio-caudal (**a**) and medio-lateral (**b**) mammograms show benign microcalcifications (arrows) in the upper-external quadrant of the left breast of a 54 years-old woman.


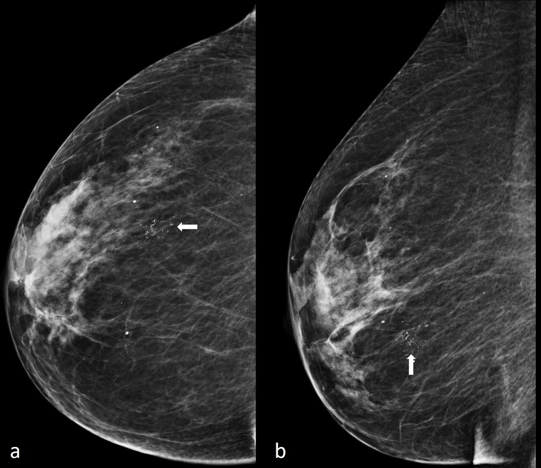


**Supplementary Fig. S2.** Example of malignant microcalcifications. Cranio-caudal (**a**) and medio-lateral (**b**) mammograms show suspicious microcalcifications (arrows) in the lower-external quadrant of the right breast of a 60 years-old woman. A vacuum-assisted breast biopsy was performed under stereotactic guidance and the histological exam results were invasive ductal carcinoma.
